# Classification of aortic stenosis using conventional machine learning and deep learning methods based on multi-dimensional cardio-mechanical signals

**DOI:** 10.1038/s41598-020-74519-6

**Published:** 2020-10-16

**Authors:** Chenxi Yang, Banish D. Ojha, Nicole D. Aranoff, Philip Green, Negar Tavassolian

**Affiliations:** 1 School of Instrument Science and Engineering, Southeast University, Nanjing, China; 2 Department of Electrical and Computer Engineering, Stevens Institute of Technology, Hoboken, NJ 07030 USA; 3 Yeshiva University, New York, NY 10032 USA; 4 Columbia University Medical Center, New York, NY 10032 USA

**Keywords:** Valvular disease, Biomedical engineering

## Abstract

This paper introduces a study on the classification of aortic stenosis (AS) based on cardio-mechanical signals collected using non-invasive wearable inertial sensors. Measurements were taken from 21 AS patients and 13 non-AS subjects. A feature analysis framework utilizing Elastic Net was implemented to reduce the features generated by continuous wavelet transform (CWT). Performance comparisons were conducted among several machine learning (ML) algorithms, including decision tree, random forest, multi-layer perceptron neural network, and extreme gradient boosting. In addition, a two-dimensional convolutional neural network (2D-CNN) was developed using the CWT coefficients as images. The 2D-CNN was made with a custom-built architecture and a CNN based on Mobile Net via transfer learning. After the reduction of features by 95.47%, the results obtained report 0.87 on accuracy by decision tree, 0.96 by random forest, 0.91 by simple neural network, and 0.95 by XGBoost. Via the 2D-CNN framework, the transfer learning of Mobile Net shows an accuracy of 0.91, while the custom-constructed classifier reveals an accuracy of 0.89. Our results validate the effectiveness of the feature selection and classification framework. They also show a promising potential for the implementation of deep learning tools on the classification of AS.

## Introduction

Heart diseases are one of the leading health hazards around the world, accounting for more than 17.9 million deaths per year in 2015, a number that is expected to grow to more than 23.6 million by 2030^1^. Among the different categories of cardiovascular diseases (CVD), valvular heart diseases (VHD) are one of the most common, accounting for up to 20% of all cardiac surgical procedures in the United States^2^.

The heart has four valves. These are the tricuspid valve, the mitral valve, the pulmonic valve, and the aortic valve^2^. Two major types of anomalies are possible in these valves. Stenosis is the narrowing of the valve which prevents an adequate outflow of blood, and insufficiency (regurgitation) is the inability to stop the backflow of the blood^3^. Aortic stenosis (AS), the stenosis of the aortic valve, is the most prevalent of all the VHDs^4^. Current methods for detecting AS involve strictly clinical procedures such as computed tomography scan, magnetic resonance imaging, and echocardiography^5^. These techniques are expensive and constraining, and not useful for continuous monitoring of subjects outside the clinic.

Wearable, noninvasive modalities can be promising in addressing the need for an out-of-the-clinic device. For instance, impedance cardiography (ICG) has been studied for evaluating the health status of heart valves^6,7^. Cardio-mechanical signals, the vibrations created by heart activities that propagate to the chest wall, are another category of modalities that have been used in the literature. Cardio-mechanical signals can be measured using micro-electromechanical system (MEMS) accelerometers and gyroscopes^8,9^. The accelerometer measures the seismo-cardiogram (SCG) signal^10,11^, while the gyroscope collects the gyro-cardiogram (GCG) signal^12–14^. These modalities have been used for the classification of several types of CVDs, including coronary artery disease^15,16^, myocardial infarction^9,17^, atrial fibrillation^18,19^, and heart failure^20^.

Our group has previously studied the capability of detecting CVDs based on time-frequency features extracted from seismo- and gyro-cardiograms. In particular, we classified general heart abnormality in^21^. In another study^22^, we conducted a binary classification between AS patients and healthy subjects and a multi-class classification among AS patients with different co-existing diseases. Statistical and morphological features were extracted from continuous wavelet transform (CWT) and empirical mode decomposition. The features were analyzed using analysis of variance (ANOVA) and evaluated using conventional machine learning (ML) algorithms, including decision tree, random forest, and neural network. Our results validated the feasibility of using a conventional machine learning framework for the classification of AS^22^.

The machine learning framework of our previous work could be further improved and investigated in several aspects. Firstly, feature selection with ANOVA might introduce bias during training because the data which are left out for testing the classifiers are included during the ANOVA process. Additionally, only one dimension is considered for each of the seismo-cardiogram (SCG) and gyro-cardiogram (GCG) modalities. Lastly, the demographic status, especially the age of the subjects, is significantly different between the healthy and AS patient groups. This might introduce demographic bias to the machine learning tasks.

In this paper, we provide point-to-point improvements in all the above aspects. A feature analysis based on Elastic Net is utilized for feature selection. This adds interpretability of the important predictors/features and removes the selection bias. Furthermore, we include SCG and GCG signals from all dimensions. The dataset has also been reformed with a control group of subjects with a similar age distribution to the AS patients. Moreover, we implemented parameter tuning based on Grid Search to optimize the performance of the ML algorithms. In addition to the three classifiers used in^22^, an extreme gradient boosting (XGBoost) method is also included to compare performances among different ML algorithms more comprehensively.

Finally, we evaluated the direct use of CWT coefficients as images for a deep-learning framework based on image classification algorithms. This method was inspired by research works in analyzing electrocardiogram (ECG) and electroencephalogram (EEG) signals. A pre-trained VGGNet model was used for the classification of ECG signals in^23^. In^24^, the use of CNN classification of motor imagery EEG based on the CWT plot was introduced to detect left/right arm movements. These preliminary studies show that temporal signals could be converted into time-frequency domain images for image-classification frameworks. Our deep learning framework is based on a 2-dimensional convolution neural network. To the best of our knowledge, this is the first image classification CNN framework based on cardio-mechanical modalities.

## Methods

**Figure 1:**
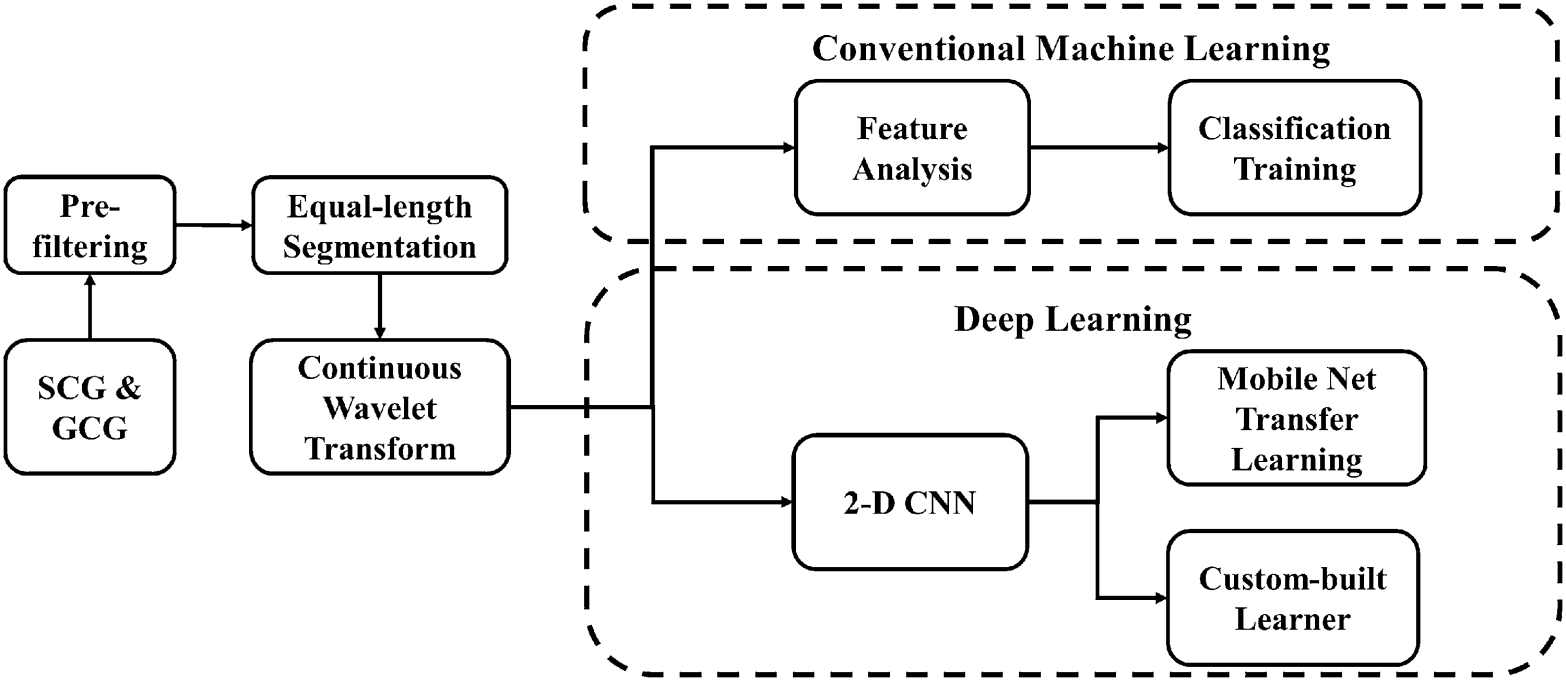
Overall signal-processing and machine learning workflow.

Figure 1 illustrates the overall workflow of signal processing and machine learning implementation. Firstly, the raw signals are pre-filtered to remove noise and focus on informative bandwidths. Next, segmentation is performed to obtain fixed-length 10-s signals. The selection of 10 seconds was based on literature^17^, as well as our own evaluations by considering various lengths from 2 to 12 s. All segmented signals were subsequently processed using continuous wavelet transform (CWT). The output from each segment are the CWT coefficients, which form a matrix with complex numerical values. For the conventional ML models, a set of statistical features were extracted from the CWT coefficient matrix and fed into various models for evaluation. For each segment, the CWT coefficients were also converted into an image. Two different convolutional neural networks were used to evaluate the images. The details of each processing step are described below.

### Data description

Twenty-one AS patients and thirteen subjects labeled as non-AS were included in this study. The AS dataset includes eleven male and ten female patients, which is identical to the AS cohort in our previous study^22^. The diagnosis of aortic stenosis was made based on 2-dimensional and Doppler transthoracic echocardiography, using the standard criteria as described in^25^. Some key parameters are measured to assist the determination of aortic stenosis conditions, including Aortic Valve Area, Aortic Velocity, and Mean Pressure Gradient. The statistical values of these parameters from AS patients are presented in Table 1. Moreover, the average age of AS patients is 86.90 years and the standard deviation is 8.43 years. A non-AS cohort which has six male subjects and seven female subjects is selected from the patient database used in^21^. The average age of the non-AS cohort is 68.38 years, with a standard deviation of 17.63 years. The subjects in this cohort are diagnosed with other types of CVDs and are confirmed to not have aortic stenosis based on a qualitative evaluation of their echocardiographic recordings. Therefore, the echocardiographic parameters from non-AS patients are not available. The demographic and echocardiographic summary of the AS and non-AS cohorts are shown in Table  1.

**Table 1:** Demographic and echocariographic information of subjects. (average ± standard deviation).

Category	Age (years)	Height (cm)	Weight (kg)
AS	86.90 ± 8.43	162.47 ± 11.30	72.31 ± 13.71
Non-AS	68.38 ± 17.63	159.31 ± 35.62	65.94 ± 4.01

Category	Aortic valve area (cm^2^)	Aortic velocity (m/s)	Mean pressure gradient (mmHg)
AS	0.75 ± 0.19	3.85 ± 0.76	34.40 ± 13.55
Non-AS	N/A	N/A	N/A

Cardio-mechanical signals were collected using a commercial wearable sensor node (Shimmer 3 from Shimmer Sensing). A three-axis MEMS accelerometer measures SCG, and a three-axis gyroscope measures GCG. Each cardio-mechanical signal has three dimensions of data: the x-axis corresponding to the shoulder-to-shoulder direction, the y-axis corresponding to the head-to-toe direction, and the z-axis corresponding to the dorso-ventral direction. In this paper, the dimensional letters X, Y, Z appended to SCG/GCG will denote the signal from the corresponding axis. For instance, SCG_Z defines the SCG signals from the z-axis.

All the data were collected at the cardiac care unit of the Columbia University Medical Center (CUMC) in collaboration with Dr. Philip Green and his colleagues. During each measurement, the subjects were asked to sit at rest on a bed or chair for at least 5 mins. The subjects breathed naturally without controlling their breathing depths. The patient experimental protocol was approved by the Institutional Review Board of CUMC under protocol number AAAR4104. All methods were carried out in accordance with relevant guidelines and regulations. The participants provided written informed consent to take part in the study. More details of the demographic information and the experimental protocol could be found in our previous studies^21,22^. Two classification tasks were evaluated in this study. The first is a binary classification between AS patients and non-AS patients. All conventional ML and CNN algorithms are tested with this task. The second task is a multi-class classification among AS patients with three different co-existing diseases, namely mitral insufficiency (MI), mitral stenosis (MS), and tricuspid regurgitation (TR). Hence, four classes of AS+MI, AS + MS, AS+TR, and AS-only are considered for multi-class classification. Only conventional ML algorithms are studied in this task.

### Pre-processing signals

The time-series signals are processed using Python language with scipy library. The signals were passed through an infinite impulse response (IIR) bandpass filter from 0.8 to 25 Hz.

After this step, the segments were created with continuous 10-s lengths using a median cutoff approach. A root mean square (RMS) filter with a step size of 500 ms was used for segmentation, and a threshold of 1.5 times the median of the RMS value was implemented to select low-noise segments. The detail for this signal processing and filtering is included in our previous study in^22^. As a result, a total of 910 segments were created for all axes and modalities.

### Time-frequency conversion of temporal signals

Each of those segments was passed through CWT using Morse wavelet as the mother wavelet. The wavelet parameters are consistent with our previous study^22^. For conventional ML analysis, the statistical features including the average (MEAN), median (MED), maximum (MAX), standard deviation (STD), and inter-quartile range (IQR) values in each frequency position along time were considered. These features are selected from a frequency range of 0.79 to 25.39 Hz and a time range of 0 to 10 s.

There are two main differences in this step between our current and previous frameworks^22^. The first difference is that more dimensions from SCG and GCG recordings have been included in feature generation. This allows for a more comprehensive view of the feature distributions and the selection of valuable features from all the axes to improve the classification performance. Secondly, we did not use the empirical mode decomposition in the current study. The reason is that much fewer features were selected from EMD compared to CWT in^22^. The ranks of the EMD-based features were also relatively low in ANOVA tests. Since EMD requires high computational power, we did not consider it to be an efficient source of feature generation. Consequently, we focus on features from CWT in this study.

For CNN algorithms, the CWT coefficients are directly used as inputs.

### Algorithm evaluation

As can be seen from Fig. 1, the workflow separates into two branches after the generation of CWT coefficients. The first branch is conventional ML algorithms, which consists of a feature analysis step and a training/validation step. The second branch is 2-D CNN implementation with two methods.

#### Conventional ML algorithms

We used Elastic Net for feature selection^26^. Elastic Net is an efficient shrinkage regularization and feature selection method that produces a low-dimension feature set without loss of significant information that classifies the original database. It has been used in genetic research as the data structure in the genetic domain has a large feature set in comparison to the available observations^26^.

For comparison purposes, we evaluated the feature selection method using four algorithms: decision tree (DT), random forest (RF), conventional multi-layer perceptron neural network (NN), and extreme gradient boosting (XGBoost). These algorithms represent four different aspects of conventional ML methods, namely simple methods (decision tree), ensemble methods (random forest), multi-layer approaches (neural network), and gradient boosted algorithms (XGBoost)^27^.

For the selection of the hyperparameters of the models, we performed the Grid Search method^28^. This method sweeps the parameters of the ML algorithms mentioned above and selects the best choices. The selected parameters are then used for validation and testing. The ML algorithms are tested in both binary and multi-class classification tasks.

#### Convolutional neural network

**Figure 2:**
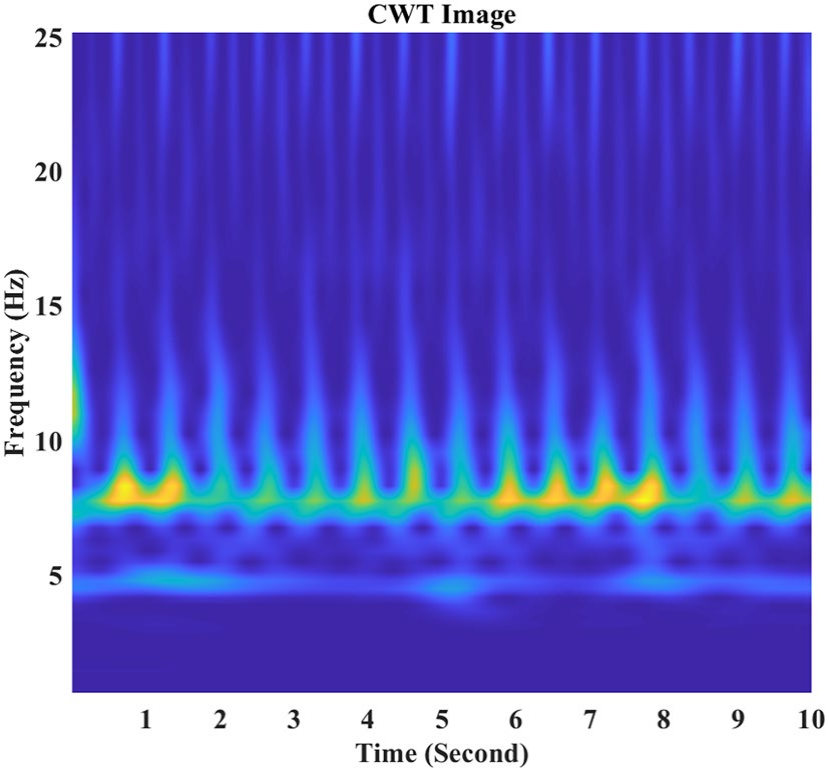
An image example of CWT coefficient heatmap generated via MATLAB.(Version: R2018b,Link: https://www.mathworks.com/products/matlab.html).

Images from the normalized power distribution of CWT coefficients were generated for CNN. An example of the CWT image output is shown in Fig. 2. In this figure, the horizontal axis represents the time domain of the segment, and the vertical axis shows the frequency domain of the CWT coefficient distribution. The values of the CWT coefficients are converted into red-green-blue (RGB) colors using a 128-point Parula color map, which is a color axis that can reveal subtle differences between values^29^.

The constructed custom-built CNN architecture is summarized in Table 2. This architecture captures the important patterns by varying kernel sizes for the convolution and pooling layers^30^. To resolve the complexity of tuning parameters, pre-trained Mobile Net architecture was also used for training to make a comparison. Mobile Net CNN is a smaller and faster architecture based on depth-wise separable convolutions. Images were resized to 224 by 224 pixels to match the input requirement of Mobile Net. The output layer was ignored from built-in Mobile Net. Instead, we appended three more layers, which are two fully connected layers and a final sigmoid layer for binary classification. It is worth mentioning that the dimensional number of the CNN architecture does not correspond to the cardiac motion dimension. The images are generated from 1-dimensional motion signals from cardiac activities to reveal the pattern differences that might not be directly discovered from the 1-dimensional time-series signals.

**Table 2:** The architecture of the CNN.

Layer (type)	Output shape	Parameters numbers
Conv2D	(None, 798, 598, 5)	50
Activation	(None, 798, 598, 5)	0
Conv2D	(None, 797, 597, 5)	105
Activation	(None, 797, 597, 5)	0
MaxPooling2	(None, 398, 298, 5)	0
Conv2D	(None, 396, 296, 5)	230
Activation	(None, 396, 296, 5)	0
Conv2D	(None, 392, 292, 5)	630
Activation	(None, 392, 292, 5)	0
MaxPooling2	(None, 78, 58, 5)	0
Flatten	(None, 22,620)	0
Dense	(None, 256)	5,790,976
Activation	(None, 256)	0
Dense	(None, 32)	8224
Activation	(None, 32)	0
Dense	(None, 1)	33
Activation	(None, 1)	0
Total parameters: 5,800,248		
Trainable parameters: 5,800,248		
Non trainable parameters: 0		

There are three different dimensions for each SCG and GCG signal. All sources and dimensions are individually evaluated via the CNN framework with the same configuration parameters. The loss type is set to binary cross-entropy. The optimizer is configured as Adam with a learning rate of 0.0008. Validation is made through the accuracy scores obtained with ten-fold cross-validation and a completely left-data-out test set with a leave-out ratio of 0.2. This CNN framework is used for evaluating the binary classification task.

## Experimental results

### Feature analysis and selection results for conventional ML methods

**Figure 3:**
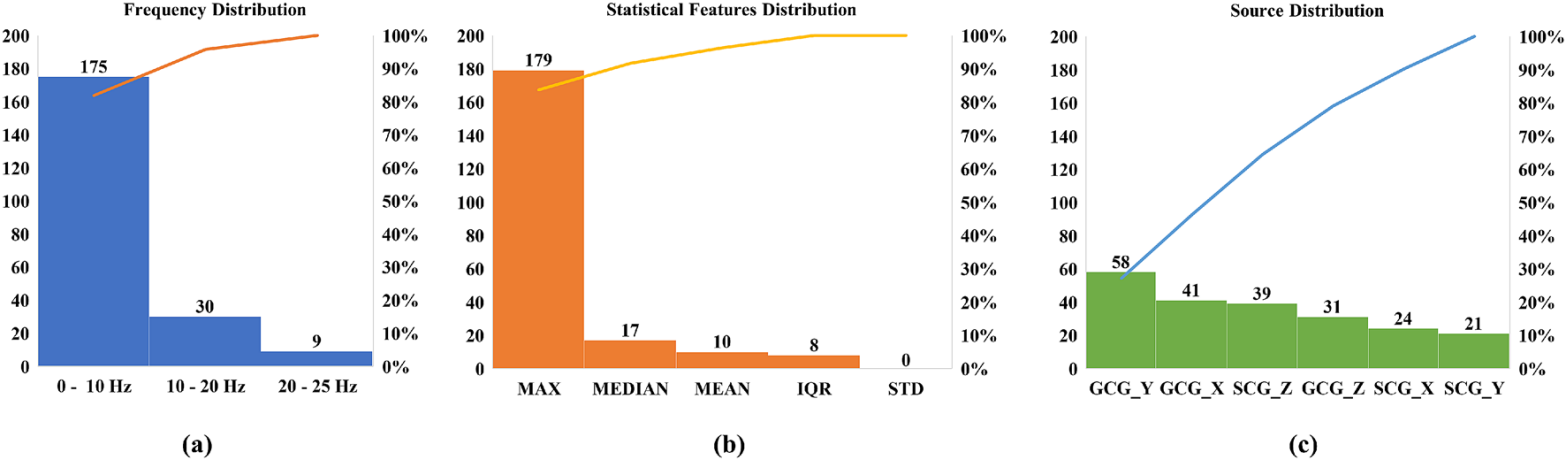
Distribution of selected features for binary classification on (**a**) frequency distribution; (**b**) statistical features distribution; and (**c**) signal source distribution (the y-axis on the left denotes bar value and the y-axis on the right denotes percentage for pareto curve; *MED* median, *IQR* inter-quartile range, *STD* standard deviation).

A total of 4764 features from 6 axes of SCG/GCG signals are analyzed using Elastic Net. A total of 214 features are selected for the binary classification between AS and non-AS, which is a reduction of feature dimension by 95.47%. The distribution of the feature selection is presented in Fig. 3. In each subfigure, the left y-axis denotes the values of the bar plot categories, while the axis on the right shows the percentage value of the Pareto curve. The Pareto curve is the accumulating percentage of the number of features that belong to a certain category compared to the total number of features. The incremental contribution of each category can be observed as the slope variations of this curve.

The frequency distribution of the features is shown in Fig. 3a. It is seen that 175 out of the 214 features were below 10 Hz, reporting a percentage of 81.78%. Between 10 and 20 Hz, there are 30 features, contributing 14.02% of the whole feature set. 9 features are found above 20 Hz, i.e., 4.20% of the total features. Our results reveal that the dominant features are below the acoustic frequency (20 Hz), especially below 10 Hz.

The statistical category distribution is demonstrated in Fig. 3b. It shows that 83.64% of the selected features are from MAX statistics. The MEDIAN, MEAN, and IQR account for the rest of the features. There are no features selected from STD. Moreover, the signal source distribution in Fig.  3c shows that GCG_Y provides the highest number of features among all signals, reporting 58 out of 214 with a percentage value of 27.1%. Combined with GCG_X and SCG_Z, the top three signal sources offer 64.48% of the total selected features. It can be observed that unlike frequency and statistical distributions, there is no dominant category in signal sources. On average, each source provides 35.6 features, suggesting that all signals contain valuable information for the binary classification.

**Figure 4:**
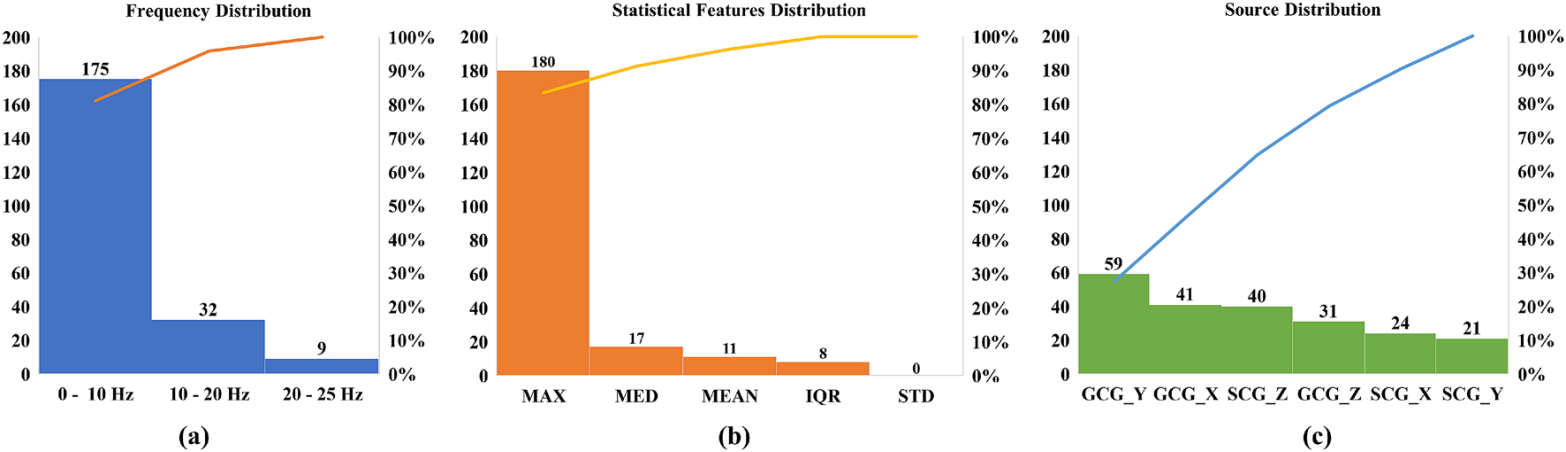
Distribution of selected features for multi-class classification on (**a**) frequency distribution; (**b**) statistical features distribution; and (**c**) signal source distribution (the y-axis on the left denotes bar value and the y-axis on the right denotes percentage for pareto curve; *MED* median, *IQR* inter-quartile range, *STD* standard deviation).

Similarly, the feature distribution results for multi-class classification are summarized in Fig. 4. A total of 216 features were extracted, reporting a reduction of 95.47% in feature dimensions. Furthermore, similar distributions could be observed compared to the distributions results of binary classification. The dominant frequency component is below 10 Hz, providing 175 features with a percentage of 81.01%. 180 features are from maximum statistics, representing 83.33% of the selected features. GCG_Y, GCG_X, and SCG_Z are still the top three signal sources, with 59, 41, and 40 features respectively. Although the distribution shows high similarity, the feature-to-feature overlap is not as high. 150 features are the same between binary and multi-class classification features.

Compared to the ANOVA results from our previous study^22^, the frequency and statistical distributions show similar observations. The significant CWT features from^22^ are below 10 Hz. In addition, 13 out of 22 features from SCG and 18 out of 27 features from GCG are based on maximum statistics, which are percentage values of 59.01% and 66.67% respectively. The percentage of maximum-based features are higher in this study, with values of more than 80%. Since^22^ only uses single-axis SCG and GCG signals, there is no fair comparison between source distributions. The differences in frequency and statistical distributions might be caused by the removal of age bias and the inclusion of multi-dimensional SCG and GCG recordings.

In summary, our investigation with an Elastic Net framework reveals the significant components for AS classification. The effectiveness of this feature selection is evaluated in the following subsection.

### Results from conventional machine learning algorithms

Tables 3 and 4 summarize the classification results of all conventional methods with the full feature set from all signal sources. The shaded values are without feature selection, and the non-shaded values are with feature selection. The results are presented in standard precision, recall, F1-score, and accuracy as follows.

1 $$\begin{aligned} Precision= \, & {} TP/(TP+FP), \end{aligned}$$

2 $$\begin{aligned} Recall= \, & {} TP/(TP+FN), \end{aligned}$$

3 $$\begin{aligned} F1\ score= \, & {} (2*Precision*Recall)/(Precision+Recall), \end{aligned}$$

4 $$\begin{aligned} Accuracy= \, & {} (TP+TN)/(TP+FN+TN+FP), \end{aligned}$$

where TP, FP, TN, and FN are true positives, false positives, true negatives, and false negatives accordingly.

**Table 3:** Results of binary classification between AS and Non-AS subjects.

Model	AS			Non-AS			Accuracy	SCG_Z only accuracy	GCG_Y only accuracy
	Precision	Recall	F1-score	Precision	Recall	F1-score			
Decision tree	*0.80*	*0.80*	*0.80*	*0.79*	*0.77*	*0.78*	*0.80*	*0.73*	*0.76*
	0.80	0.79	0.79	0.78	0.76	0.75	0.78	N/A	
Random forest	*0.83*	*0.83*	*0.82*	*0.83*	*0.78*	*0.79*	*0.81*	*0.69*	*0.69*
	0.83	0.83	0.82	0.83	0.78	0.79	0.81	N/A	
Neural network	*0.68*	*0.76*	*0.71*	*0.68*	*0.57*	*0.61*	*0.69*	*0.69*	*0.78*
	0.68	0.76	0.71	0.67	0.57	0.61	0.68	N/A	
XGBoost	***0.95***	***0.94***	***0.95***	***0.94***	***0.95***	***0.94***	***0.93***	***0.75***	***0.86***
	0.94	0.94	0.94	0.94	0.95	0.94	0.94	N/A	

Italicized value for evaluations without feature selection and underlined values for evaluations after feature reduction by 95.47%.

XGBoost has the best performance among the classifiers with an average accuracy of 0.93 in bold.

**Table 4:** Results of multi-class classification of AS with co-existing VHDs.

Model	Leave-out validation														
	AS			AS + MI			AS + MS			AS + TR			Overall accuracy	SCG_Z only accuracy	GCG_Y only accuracy
	Precision	Recall	F1-score	Precision	Recall	F1-score	Precision	Recall	F1-score	Precision	Recall	F1-score			
Decision tree	*0.78*	*0.93*	*0.85*	*0.91*	*0.77*	*0.83*	*0.92*	*0.69*	*0.79*	*0.89*	*0.89*	*0.89*	*0.85*	*0.82*	*0.83*
	0.70	0.77	0.73	0.59	0.77	0.67	1.00	0.50	0.67	0.75	0.79	0.77	0.72	N/A	
Random forest	*0.94*	*1.00*	*0.97*	*1.00*	*1.00*	*1.00*	*1.00*	*0.88*	*0.93*	*1.00*	*1.00*	*1.00*	*0.97*	*0.95*	*0.93*
	0.94	1.00	0.97	1.00	1.00	1.00	1.00	0.88	0.93	1.00	1.00	1.00	0.97	N/A	
Neural network	*0.97*	*1.00*	*0.98*	*1.00*	*1.00*	*1.00*	*1.00*	*0.94*	*0.97*	*1.00*	*1.00*	*1.00*	*0.99*	*0.82*	*0.87*
	0.97	1.00	0.98	1.00	0.92	0.96	1.00	0.88	0.93	0.90	1.00	0.95	0.96	N/A	
XGBoost	*0.94*	*1.00*	*0.97*	*1.00*	*0.92*	*0.96*	*1.00*	*0.88*	*0.93*	*0.95*	*1.00*	*0.97*	*0.96*	*0.95*	*0.94*
	0.94	1.00	0.97	1.00	0.92	0.96	1.00	0.94	0.97	1.00	1.00	1.00	0.97	N/A	

*AS* only AS without co-occurrences, *AS + MI* mitral insufficiency along with AS, *AS + MS* mitral stenosis along with AS, and *AS + TR* tricuspid regurgitation along with AS.

Italicized values for evaluations without feature selection and underlined values for evaluations after feature reduction by 95.47%.

#### Binary classification

The results for binary classification are shown in Table 3. The classifiers are tested with a leave-one-subject-out validation strategy, and the best metrics from the Grid Search are reported. As noted in Table 3, XGBoost has the best performance among the classifiers with an average accuracy of 0.93. The highest precision and recall from XGBoost both report 0.95. In comparison, the decision tree gives an overall accuracy of 0.80, which is slightly outperformed by the random forest with a value of 0.81. Considering the implementation of leave-one-subject-out validation, these two classifiers report acceptable values. However, the neural network method provides much lower values with 0.68 in precision, 0.76 in recall, and 0.69 in overall accuracy.

In addition, the results without and with feature selection report marginal differences, showing that our selective feature set is effective in binary classification of AS. Accuracy differences are 0.02 from DT, 0.00 from RF, 0.01 from NN, and 0.01 from XGBoost.

#### Multi-class classification

Table 4 shows the detailed evaluation of the four classes of AS with co-occurrences of VHDs. The performance metrics by the classifiers are shown for each class individually. Due to the limited number of subjects for each class, it is not feasible to perform a statistically reasonable leave-subject-out validation. Instead, we conducted a leave-data-out validation by leaving 0.2 of all data for testing. The shaded values in the table are results with all the features, and the non-shaded values are after feature selection (reducing features by 95.47%). As can be observed in Table 4, there is no significant alteration for the performance before and after feature selection except for the decision tree classifier. The differences between with and without feature selection are 0.13, 0.00, 0.03, and 0.01 from the DT, RF, NN, and XGBoost methods respectively, suggesting a less-stable performance from the decision tree for multi-class classification. In general, feature selection has minimal influence on multi-class classification performance. All the models except DT have accuracies of more than 96%. Specifically, the precision for the pure AS class is lower than the recall, reporting 0.94 vs. 1.00 from XGBoost, 0.97 vs. 1.00 from neural network, and 0.94 vs. 1.00 from random forest. For the classes of AS with mitral insufficiency and mitral stenosis, precision identification outperforms recall identification. Furthermore, recall metrics for AS with tricuspid regurgitation are better than or comparable with the precision metrics.

In summary, the classification of VHDs can be performed by combining binary and multi-class classifiers. For general binary classification between AS and non-AS subjects, XGBoost outperforms all the other methods. For multi-class classification, different classifiers need to be selected. For instance, the random forest classifier should be chosen for the classification of AS + MI. Therefore, a combined strategy can be implemented to use XGBoost for binary classification to generally detect AS, and then use random forest to detect AS + MI.

#### Source-wise comparison for binary classification

Features from SCG_Z and GCG_Y are independently evaluated for the conventional methods with leave-one-subject-out validation. The purpose is to compare the performance of combining all axes with the performance of single-axis SCG/GCG as used in our previous study^22^. The overall accuracy numbers for binary classification are presented in Table 3. The single-axis results show lower values from decision tree, random forest, and XGBoost classifiers but not from the neural network classifier. Instead, the SCG_Z-based results report no difference with the all-axes results. In addition, the GCG_Y axis shows a higher accuracy of 0.86 in comparison with the value of 0.69 from all-axes results. Overall, the utilization of all axes shows benefits for binary classification. The performance of XGBoost with the highest accuracy of 0.93 is considerably superior when using all the axes.

The single-axis comparison for multi-class classification is shown in the rightmost columns in Table 4. The accuracy from DT reports 0.82 from SCG_Z and 0.83 from GCG_Y. In comparison, the overall accuracy is 0.85 when including all the axes. The RF classifier shows 0.95 and 0.93 from SCG_Z and GCG_Y, respectively. In comparison, the accuracy using all the axes is 0.97. A marginal difference is observed from XGBoost classifier, with values of 0.01 (0.95 vs. 0.96) and 0.02 (0.94 vs. 0.96) respectively from SCG_Z and GCG_Y. However, more significant differences can be found for the NN classifier. The SCG_Z shows an accuracy of 0.82, which is outperformed by GCG_Y with accuracy of 0.87. Both values are much lower than the result of 0.99 when using features from all the axes. In summary, the benefit of using features from all sources for multi-class classification is noticeable but not as significant as the results for binary classification.

**Figure 5:**
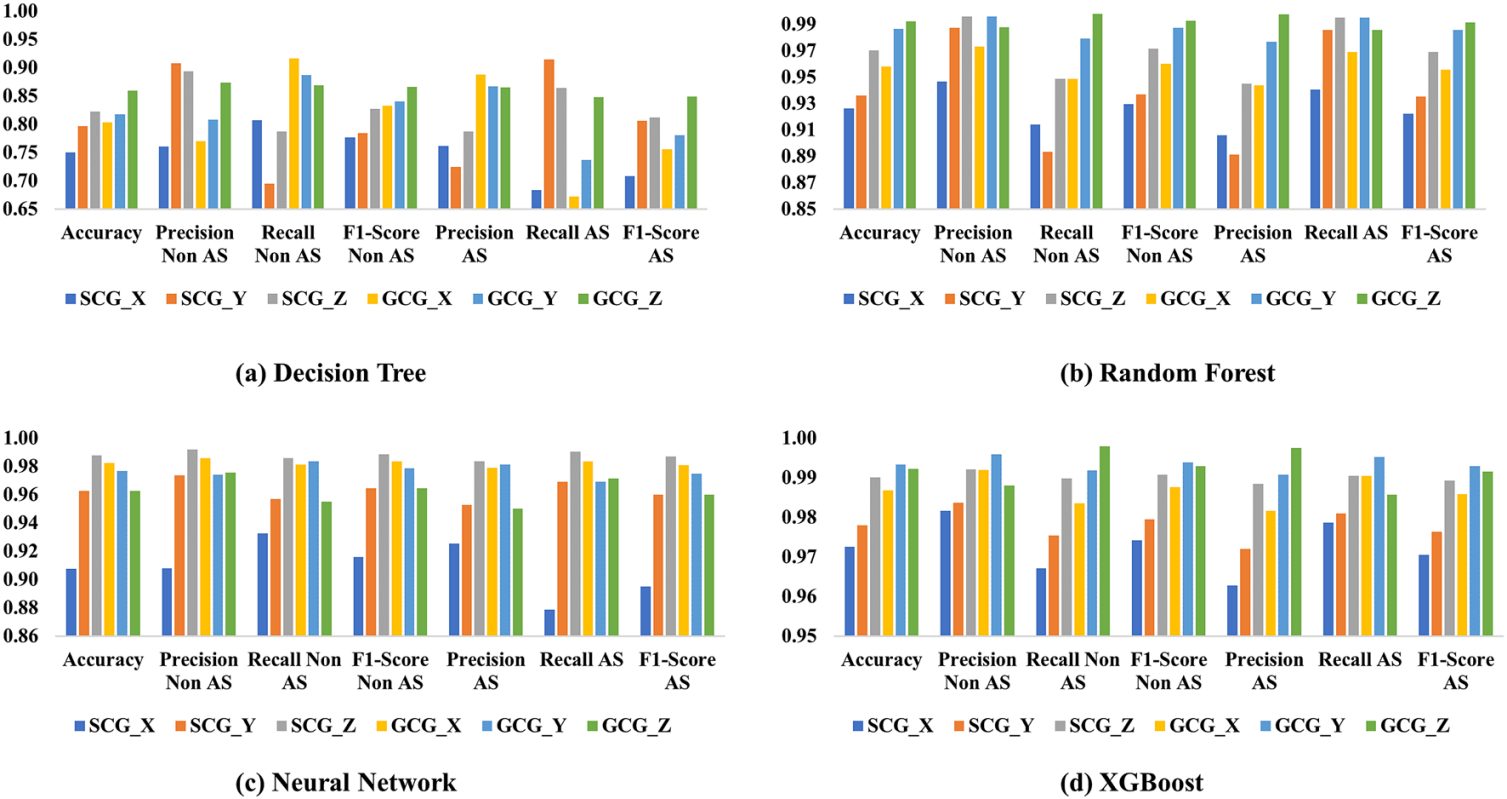
Source-specific evaluation with different metrics for (**a**) decision tree, (**b**) random forest, (**c**) multi-layer perceptron (MLP) neural network, and (**d**) XGBoost.

Figure 5 shows the results of the conventional methods with features from a single source/axis using leave-data-out validation with a ratio of 0.2. This experiment is conducted to create fair comparison metrics for the evaluation of CNN, which is also based on single-axis signal sources. It is to be noted that the leave-data-out validation is used in this test to remain consistent with the CNN method. As shown in Fig. 5a, the best performance for the decision tree method is from GCG_Z, with an accuracy of 0.85. For random forest, the best performance is from GCG_Z with accuracy of 0.99. Results from the neural network show the best performance of 0.98 on accuracy from SCG_Z, and for XGBoost, the best overall accuracy is 0.99 from GCG_Y. These metrics are higher than those from leave-one-subject-out validation, which is expected due to the inclusion of subject bias.

Overall, XGBoost shows high accuracy and stable performance without much fluctuations among all the axes and modalities, whereas the decision tree seems to be least satisfactory. The neural network generally shows comparable performance to the random forest.

### Results from 2-D CNN

**Figure 6:**
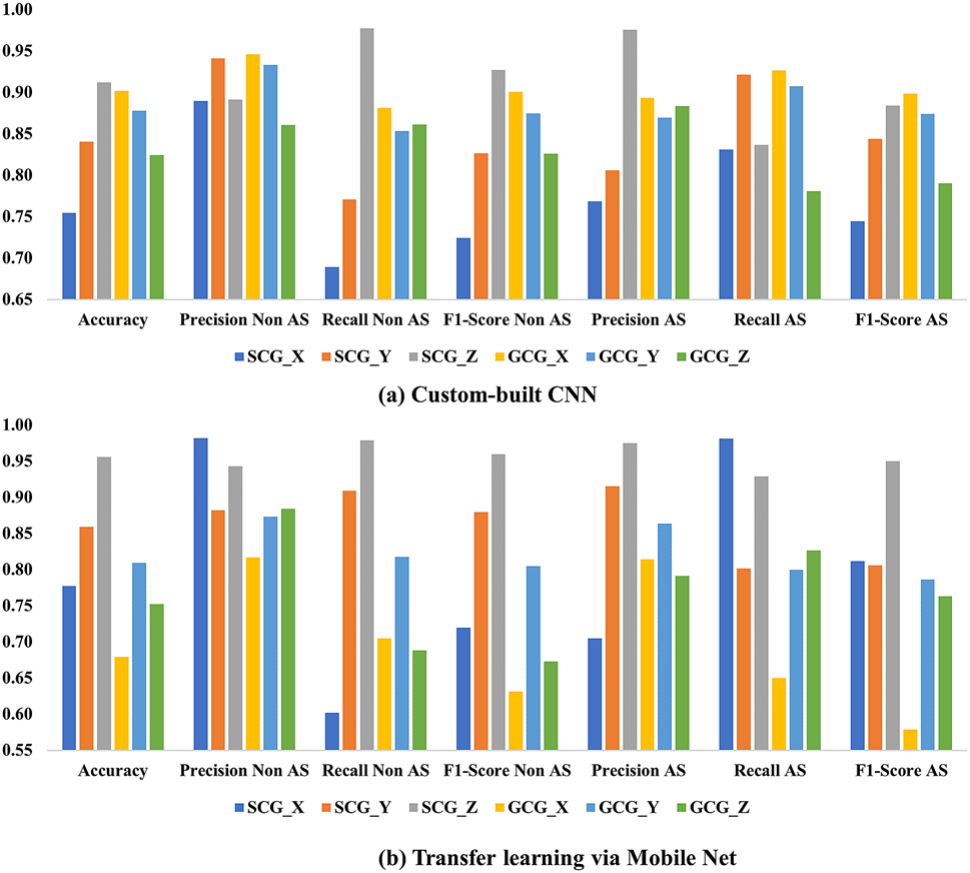
Evaluation for 2-dimensional conventional learning (2-D CNN) via (**a**) custom-built CNN and (**b**) Mobile Net (transfer learning).

The CNN model was constructed from the root level using the architecture shown in Table 2 as well as using the transfer learning approach via Mobile Net architecture. Figure 6 summarizes the classification results based on CNN from both custom-built CNN and the pre-trained Mobile Net classifier. The summary of the results obtained with single-axis, single-modality datasets using the custom-built CNN is presented in Fig. 6a. It is seen that the SCG_Z axis reports the highest overall accuracy of 0.91. Specifically, the recall of the non-AS class outperforms precision of the same class with 0.98 vs. 0.89. On the other hand, the precision of the AS class is 0.97, higher than the recall value of 0.84.

Figure 6b illustrates the results from transfer learning using Mobile Net. The best overall accuracy is 0.95 from SCG_Z. Furthermore, the precision of non-AS shows 0.94, which is higher than the value of 0.89 from the custom-built model. The recall of non-AS shows comparable performance, with 0.97 in comparison to 0.98 for the custom CNN. It is notable that the images from GCG_X show a very low accuracy of 0.67, suggesting a nonstable performance among signal sources. Overall, the average accuracy among all the axes is 0.85 from the custom-built CNN and 0.80 from the Mobile Net. In comparison, the average results in Fig. 5 report 0.81 from decision tree, 0.96 from random forest, 0.96 from neural network, and 0.98 from XGBoost. In general, the CNN framework shows comparable performance with the conventional ML method with the worst metrics.

Although the CNN framework does not perform as well as the conventional method, it eliminates the need for feature generation and analysis, providing a simpler framework that directly uses the CWT coefficients as images. Furthermore, the training and testing of CNN networks generally requires more data than conventional methods. Therefore, the accuracy could be improved by collecting a larger database of subjects. Combining CWT images from multiple axes could also improve the accuracy^23^.

### Comparison with other works

Table 5 compares our results with results from the literature, including our previous work^22^. For the binary classification with conventional ML algorithms, it can be seen that the values in^22^ are higher in all metrics. However, it is to be noted that the results of^22^ are biased in terms of feature and age. Our results are comparable with the results of^17^ in terms of precision, which was validated via leave-subject-out method. In addition, the recall values of this work are higher than those of^17^. In summary, our results further support the feasibility of this method in the binary classification of AS.

**Table 5:** Performance comparison with other research methods.

Methods	Target CVD(s)	Classifier(s)	Accuracy	Precision	Recall	Validation method	References
**Binary classification**							
SCG + GCG	Acute Myocardial Infarction	Kernel SVM	N/A	0.95	0.82	Leave-subject-out	^20^
SCG + GCG	Aortic Stenosis (AS)	RF	0.98	0.99	0.98	Leave-subject-out	^22^
Proposed method	**AS**	**XGBoost**	**0.93**	**0.95**	**0.95**	Leave-subject-out	
**Multi-class classification**							
ICG	25 CVD classes	Discriminant analysis	0.95	N/A	N/A	Leave-data-out	^6^
SVM with ICG	5 VHD classes	SVM and KNN	0.98	1.00	0.97	Leave-data-out	^7^
SCG + GCG	4 AS classes	RF	0.96	0.97	0.97	Leave-data-out	^22^
Proposed method	**4 AS classes**	**XGBoost**	**0.97**	**0.99**	**1.00**	Leave-data-out	
**CWT-based CNN classification**							
ECG	5 ECG types	VGGNet	0.99	1.00	0.96	Leave-data-out	^23^
EEG	N/A	CNN	0.78	N/A	N/A	Leave-data-out	^24^
SCG/GCG	**AS**	**Mobile Net**	**0.95**	**0.97**	**0.94**	Leave-data-out	

Regarding the multi-class classification, the new results show marginal improvement compared to the results from our previous work^22^. The accuracy, precision, and recall are all slightly higher compared to the values in^22^. In addition, the general performance is higher than^5^ and has closer performance to^7^. In conclusion, there is no significant benefit in multi-class classification based on the current database. A larger database for each class could further evaluate the framework in this aspect.

This work is the first CNN image classification framework for cardio-mechanical signals. Therefore, there are no other studies to provide a fair comparison. Compared with the two studies that motivated this research, the overall accuracy is higher than the work in EEG classification^24^, but lower than the results from the ECG classification study^23^. The ECG classification algorithm was trained with a database with 175,000 beats from 47 subjects, suggesting the potential benefit of an extensive database.

## Conclusion and future work

Firstly, this paper presents an improved conventional machine learning framework for AS classification. The framework generates unbiased feature selection and classification results. The statistical feature analysis results reveal that the dominant features are below 10 Hz in frequency and mostly from maximum features. Both the feature selection and classification results indicate that it is beneficial to use multi-dimensional SCG and GCG recordings for AS classification. The conventional methods show excellent performance for the classification of AS and non-AS subjects with accuracies of up to 0.97. It is also shown that a gradient boosted method such as XGBoost performs better than other algorithms. An image classification framework based on CNN is also evaluated. Feature identification, extraction, and selection can be avoided using this approach. The 2-D CNN results are slightly inferior to the results of the conventional machine learning algorithms. The CNN framework shows a promising potential to be further improved.

This research can be improved by collecting data from a larger number of subjects. Both the AS and non-AS cohorts should be extended with a wide variety of demographic conditions. The inclusion of more data allows the application of leave-subject-out validation method to both conventional multi-class classification and the binary CNN-based classification. This could reduce subject bias, allowing a more comprehensive evaluation of the classification frameworks. A large number of subjects will also benefit the generalization capability and facilitate deeper insights into the models. For instance, the correlation between features from cardio-mechanical signals and echocardiographic parameters can be evaluated from continuous echocardiogram recordings of a large number of subjects.

Based on the pilot findings of this work, the time-domain, one-dimension cardio-mechanical signals can be converted into 2-D observations and demonstrate image-like characteristics. Therefore, the well-developed feature extraction and machine learning methods used in cardiac imaging^31^ and finite element analysis of the cardiovascular bio-mechanics^32^ reveal promising potential for the analysis of cardio-mechanical signals.

## Data Availability

Data are available on request due to privacy or other restrictions.

## Code availability

All Python scripts were created in python version 3.6.8. Specific parameters and packages used to generate, test, and process the current dataset are mentioned in detail in the “Methods” section.

## References

[1] Benjamin, E. J. *et al.* Heart disease and stroke statistics–2018 update: a report from the american heart association. *Circulation* **137**, 10.1161/cir.0000000000000558 (2018). 10.1161/CIR.0000000000000558 29386200

[2] Maganti K, Rigolin VH, Sarano ME, Bonow RO (2010). Valvular heart disease: diagnosis and management. Mayo Clin. Proc..

[3] Klabunde R (2011). Cardiovascular Physiology Concepts.

[4] Carabello BA, Paulus WJ (2009). Aortic stenosis. Lancet.

[5] Faggiano P (2012). Prevalence of comorbidities and associated cardiac diseases in patients with valve aortic stenosis. Potential implications for the decision-making process. Int. J. Cardiol..

[6] Salah RB, Alhadidi T, Mansouri S, Naouar M (2015). A new method for cardiac diseases diagnosis. Adv. Biosci. Biotechnol..

[7] Chabchoub S, Mansouri S, Ben Salah R (2018). Detection of valvular heart diseases using impedance cardiography icg. Biocybern. Biomed. Eng..

[8] D’Mello, Y. *et al.* Real-time cardiac beat detection and heart rate monitoring from combined seismocardiography and gyrocardiography. *Sensors* **19**, 3472. 10.3390/s19163472 (2019). 10.3390/s19163472 PMC6719139 31398948

[9] Inan, O. T. Wearable sensing of left ventricular function. *Mobile Health* **265–287**, 10.1007/978-3-319-51394-2_14 (2017).

[10] D’Mello, Y. *et al.* Real-time cardiac beat detection and heart rate monitoring from combined seismocardiography and gyrocardiography. *Sensors* **19**, 3472 (2019). 10.3390/s19163472 PMC6719139 31398948

[11] Yang C, Tavassolian N (2018). Combined seismo- and gyro-cardiography: a more comprehensive evaluation of heart-induced chest vibrations. IEEE J. Biomed. Health Inform..

[12] Taebi A, Mansy HA (2017). Time-frequency distribution of seismocardiographic signals: a comparative study. Bioengineering.

[13] Jafari Tadi, M. *et al.* Gyrocardiography: a new non-invasive monitoring method for the assessment of cardiac mechanics and the estimation of hemodynamic variables. *Sci. Rep.* **7**, 10.1038/s41598-017-07248-y (2017). 10.1038/s41598-017-07248-y PMC5533710 28754888

[14] Inan OT (2014). Ballistocardiography and seismocardiography: a review of recent advances. IEEE J. Biomed. Health Inform..

[15] Korzeniowska-Kubacka I, Bilinska M, Piotrowicz R (2005). Usefulness of seismocardiography for the diagnosis of ischemia in patients with coronary artery disease. Ann. Noninvas. Electrocardiol..

[16] Tavakolian, K., Blaber, A., Akhbardeh, A., Ngai, B. & Kaminska, B. Estimating cardiac stroke volume from the seismocardiogram signal. *CMBES Proc.* **33**, (2010).

[17] Lahdenoja, O. *et al.* A smartphone-only solution for detecting indications of acute myocardial infarction. In *2017 IEEE EMBS International Conference on Biomedical Health Informatics (BHI)*, 197–200 (2017).

[18] Iftikhar, Z. *et al.* Multiclass classifier based cardiovascular condition detection using smartphone mechanocardiography. *Sci. Rep.* **8**, 10.1038/s41598-018-27683-9 (2018). 10.1038/s41598-018-27683-9 PMC6008477 29921933

[19] Hurnanen T (2017). Automated detection of atrial fibrillation based on timeâfrequency analysis of seismocardiograms. IEEE J. Biomed. Health Inform..

[20] Inan, O. T. *et al.* Novel wearable seismocardiography and machine learning algorithms can assess clinical status of heart failure patients. *Circulation* **11**, 10.1161/circheartfailure.117.004313 (2018). 10.1161/CIRCHEARTFAILURE.117.004313 PMC5769154 29330154

[21] Yang, C., Aranoff, N. D., Green, P. & Tavassolian, N. A binary classification of cardiovascular abnormality using time-frequency features of cardio-mechanical signals. In *2018 40th Annual International Conference of the IEEE Engineering in Medicine and Biology Society (EMBC)*, 5438–5441 (2018). 10.1109/EMBC.2018.8513644 30441567

[22] Yang C, Aranoff ND, Green P, Tavassolian N (2020). Classification of aortic stenosis using time-frequency features from chest cardio-mechanical signals. IEEE Trans. Biomed. Eng..

[23] Al Rahhal MM, Bazi Y, Al Zuair M, Othman E, BenJdira B (2018). Convolutional neural networks for electrocardiogram classification. J. Med. Biol. Eng..

[24] Lee, H. K. & Choi, Y. A convolution neural networks scheme for classification of motor imagery eeg based on wavelet time-frequecy image. In *2018 International Conference on Information Networking (ICOIN)*, 906–909 (2018).

[25] Nishimura RA (2017). 2017 aha/acc focused update of the 2014 aha/acc guideline for the management of patients with valvular heart disease: a report of the american college of cardiology/american heart association task force on clinical practice guidelines. J. Am. Coll. Cardiol..

[26] Romagnoni, A., Jégou, S., Van Steen, K., Wainrib, G. & Hugot, J.-P. Comparative performances of machine learning methods for classifying crohn disease patients using genome-wide genotyping data. *Sci. Rep.* **9**, 10.1038/s41598-019-46649-z (2019). 10.1038/s41598-019-46649-z PMC6637191 31316157

[27] Zou H, Hastie T (2005). Regularization and variable selection via the elastic net. J. R. Stat. Soc. B.

[28] Bergstra J, Bengio Y (2012). Random search for hyper-parameter optimization. J. Mach. Learn. Res..

[29] Nuñez JR, Anderton CR, Renslow RS (2018). Optimizing colormaps with consideration for color vision deficiency to enable accurate interpretation of scientific data. PLOS ONE.

[30] Howard, A. *et al.* Mobilenets: efficient convolutional neural networks for mobile vision applications. arXiv preprint arXiv:1704.04861 (2017).

[31] Madani A, Ong JR, Tibrewal A, Mofrad MR (2018). Deep echocardiography: data-efficient supervised and semi-supervised deep learning towards automated diagnosis of cardiac disease. NPJ Digit. Med..

[32] Madani, A., Bakhaty, A., Kim, J., Mubarak, Y. & Mofrad, M. R. Bridging finite element and machine learning modeling: stress prediction of arterial walls in atherosclerosis. *J. Biomech. Eng.* **141** (2019). 10.1115/1.4043290 30912802

